# Partial Substitution of Nitrogen Fertilizer with Biogas Slurry Increases Rice Yield and Fertilizer Utilization Efficiency, Enhancing Soil Fertility in the Chaohu Lake Basin

**DOI:** 10.3390/plants13152024

**Published:** 2024-07-23

**Authors:** Yangting Lu, Qian Xiao, Sheng Wu, Haoqiang Yuan, Tingfeng Gao, Tianpei Cai, Xiaowen Wu, Youhua Ma, Xia Liao

**Affiliations:** 1 Key Laboratory of Farmland Ecological Conservation and Pollution Prevention and Control in Anhui Province, College of Resources and Environment, Anhui Agricultural University, Hefei 230036, China; luyt0512@163.com (Y.L.);; 2 Agricultural Technique Extension Center of Lujiang County in Anhui Province, Hefei 231500, China

**Keywords:** biogas slurry, fertilizer optimization, rice yield, fertilizer utilization efficiency, soil fertility

## Abstract

To investigate the effects of biogas slurry substitution for fertilizer on rice yield, fertilizer utilization efficiency, and soil fertility, a field experiment was conducted on rice–wheat rotation soil in the Chaohu Lake Basin for two consecutive years, with the following six treatments: no fertilization (CK), conventional fertilization (CF), optimized fertilization (OF), biogas slurry replacing 15% of fertilizer (15% OFB), biogas slurry replacing 30% of fertilizer (30% OFB), and biogas slurry replacing 50% of fertilizer (50% OFB). The field experiment results showed that, compared with CF treatment, OF treatment in 2022 and 2023 significantly increased (*p* < 0.05) rice yield, promoted the uptake of nitrogen (N), phosphorus (P), and potassium (K) by grains and straws, improved fertilizer utilization efficiency, and increased the contents of soil organic C (SOC), NH_4_^+^-N, NO_3_^−^-N, hydrolysable N, and available P. The 15% OFB and 30% OFB treatments significantly increased (*p* < 0.05) rice grain and straw yields compared with CF treatment, and rice grain and straw yields were the highest in the 30% OFB treatment. Compared with CF and OF treatments, 30% OFB treatment significantly increased (*p* < 0.05) the N, P, and K uptake of grains and straws and increased the fertilizer utilization efficiency. Compared with CF treatment, the grain yield of 50% OFB treatment was significantly decreased (*p* < 0.05) in 2022, and there was no significant difference in 2023, which may be because the biogas slurry was applied before planting in 2023 to provide more nutrients for early rice growth. Compared with CF treatment, 30% OFB treatment significantly increased (*p* < 0.05) the contents of SOC, NH_4_^+^-N, available K, and hydrolysable N. In summary, optimizing N and K topdressing methods can increase rice yield and improve the fertilizer utilization efficiency and soil fertility. The 30% OFB treatment resulted in the highest rice yield, fertilizer utilization efficiency, and improved soil fertility, indicating that biogas slurry replacing 30% of fertilizer was the best application mode for rice in this region.

## 1. Introduction

Rice is an important food crop in China and plays an important role in ensuring national food security. Due to the high yield of rice, farmers tend to apply a large amount of fertilizer, which has led to some negative consequences, such as low fertilizer utilization efficiency and environmental pollution [1,2,3]. Currently, China’s fertilizer production and consumption are 3.7 times that of the world average, and the fertilizer intensity and application amount per unit area far exceed the world average [1]. The amount of synthetic fertilizer application in China has increased from7.8 × 10^4^ tons in the 1950s to 6.02 × 10^7^ tons, increasing at a rate of 9.8% per year [1]. The amount of fertilizer applied in the central region of Anhui Province ranked first among the four major regions of east, central, west, and northeast China [2]. From 2006 to 2015, the amount of fertilizer applied in Anhui Province increased by 1.4 × 10^7^ tons, with an annual growth rate of 41.41% [2]. Nitrogen (N) is an essential nutrient element for increasing crop yield and improving crop quality [3]. From 2010 to 2015, the average N fertilizer application rate in China was the highest across the world, but the N fertilizer utilization efficiency in the current season was less than 35% [4], resulting in economic losses, biodiversity reduction, and environmental pollution. Therefore, N fertilizer application technology is a difficult problem that needs to be overcome in agriculture at present [5]. A short-term effect of excessive fertilizer application was the increase in crop yield, but the long-term effects include crop yield reducing, crop quality declining, fertilizer utilization efficiency declining, soil acidification, nutrient loss, and environmental pollution [6].

With the development of animal husbandry, the discharge of livestock and poultry excrement is increasing. Without safe treatment before discharging, it is directly discharged into the environment, causing environmental pollution and ultimately endangering human health [7]. Data have shown that in 2020, Lujiang County, which belongs to Hefei City, Anhui Province, raised 1.50 × 10^5^ pigs, 7.68 × 10^5^ laying hens, and 2.44 × 10^6^ meat and poultry animals, with an annual output of 1.08 × 10^6^ tons of livestock and poultry manure [8]. China produces 3.8 billion tons of livestock and poultry manure every year, and its utilization rate is 70% [9]. However, the allocational rate of livestock and poultry manure treatment facilities is only 63% [9]. Thus, it is necessary to further improve the safe treatment and resource utilization of livestock and poultry manure [9]. In the process of livestock manure composting and biogas engineering, the N and P nutrient contents of livestock and poultry manure, were reduced by 22% and 10%, respectively, and the available N and P nutrient supply amounts were 0.9 × 10^4^ and 0.3 × 10^4^ t, respectively [10,11]. Therefore, the rational consumption and resource-oriented utilization of livestock and poultry manure is an urgent problem to be solved.

A biogas slurry is an organic liquid fertilizer produced by the anaerobic fermentation of livestock and poultry manure. It was rich in N, P, and K, as well as microelements, such as iron, calcium, and copper [12]. Partial replacement of chemical fertilizer with biogas slurry can reduce the amount of chemical fertilizer needed, enrich the soil, and enable the resource consumption of livestock and poultry manure [10]. Compared with conventional fertilizer application, replacing 25% of fertilizer with biogas slurry can increase the soil available K and available P contents, while replacing 100% of fertilizer with biogas slurry can significantly increase the soil total N, organic C, available P, and available K contents [13]. In recent years, there have been many studies on the application of biogas slurry as a fertilizer replacement for different crops. However, the effects of different biogas slurry proportions on yield and fertilizer utilization efficiency were inconsistent. Compared with conventional fertilizer application, the replacement of 15% and 30% of fertilizer with biogas slurry can significantly increase wheat yield, while the replacement of 50% of fertilizer can significantly reduce wheat yield [14]. Replacing 40% of fertilizer with biogas slurry can increase the yield of summer corn and winter wheat and improve the fertilizer utilization efficiency [15,16]. However, the replacement of 50% and 75% of fertilizer with biogas slurry increased rice yield [17]. The effects of biogas slurry replacement of fertilizer on different crops and soil nutrients were jointly determined by the biogas slurry content, replacement ratio, soil properties, climate factors, and farming systems [14]. Therefore, research on the optimal ratio of biogas slurry to replace fertilizer should be carried out in specific regions, aiming at providing a theoretical basis for biogas slurry consumption and fertilizer reduction in the region.

In this study, field experiments with biogas slurry as a fertilizer replacement were carried out in a rice–wheat rotation system in the Chaohu Lake Basin, and three ratios of biogas slurry were set to replace fertilizer (15%, 30%, and 50%) to explore the effects of different ratios of biogas slurry on rice yield, fertilizer utilization efficiency, and soil fertility. In two years of field trials, the optimal ratio of biogas slurry as a fertilizer replacement in the Chaohu Lake Basin was determined, providing a scientific basis for the quality and sustainable development of agricultural products under rice–wheat rotation in central Anhui.

## 2. Materials and Methods

### 2.1. Study Site

The field experiment was conducted from June 2022 to November 2023 in Guanghan Village, Guohe Town, Lujiang County, Hefei City, Anhui Province (31°29′ N, 117°12′ E), which belongs to the subtropical monsoon climate zone with a mild climate, abundant rainfall, and an annual average temperature of 15.9 °C. The average precipitation was 1262.9 mm. The soil was derived from alluvial sediments, and the physical and chemical properties of the 0–20 cm soil layer were as follows: soil pH, 4.83; bulk density, 1.01 g·cm^−3^; SOC, 9.79 g·kg^−1^; TN, 1.11 g·kg^−1^; NH_4_^+^-N, 9.24 mg·kg^−1^; NO_3_^−^-N, 1.56 mg·kg^−1^; hydrolysable N, 109.67 mg·kg^−1^; available P, 10.63 mg·kg^−1^; and available K, 124.67 mg·kg^−1^.

### 2.2. Field Experiment

The field experiment began on 15 June 2022, and consisted of six treatments: no fertilization (CK), conventional fertilization (CF), optimized fertilization (OF), biogas slurry replacing 15% of fertilizer (15% OFB), biogas slurry replacing 30% of fertilizer (30% OFB), and biogas slurry replacing 50% of fertilizer (50% OFB). Each treatment was performed in three repetitions. The plots were designed in a completely random block design, with an area of 30 m^2^ (5 m × 6 m), which were separated by a ridge covered with plastic film. Irrigation and drainage channels were used to eliminate the mutual interference of water and fertilizer among the plots, and protection lines were set.

The chemical fertilizers tested were urea (N ≥ 46.4%), superphosphate (P_2_O_5_ ≥ 12%), and potassium chloride (K_2_O ≥ 60%). The biogas slurry was obtained from Wugongshan Livestock and Poultry Breeding Co., Ltd., Hefei, China. The nutrient content of the biogas slurry was measured before application. The N, P_2_O_5_, and K_2_O contents were 0.15%, 0.02%, and 0.03%, respectively, before application in the 2022 rice season. In 2023, the N, P_2_O_5_, and K_2_O contents in the biogas slurry were 0.11%, 0.05%, and 0.03%, respectively.

The amount of fertilization in each treatment is shown in Table 1. P fertilizer treatment was applied as a base fertilizer, and N and K fertilizers were divided into base fertilizer and topdressing fertilizer. N fertilizer treatment for conventional fertilization was applied as 50% base fertilizer, 35% tillering fertilizer, and 15% booting fertilizer, and K fertilizer was applied as 50% base fertilizer and 50% booting fertilizer. The optimized fertilization treatment of N fertilizer was applied as 50% base fertilizer, 30% tillering fertilizer, and 20% booting fertilizer, and K fertilizer was applied as 45% base fertilizer, 15% tillering fertilizer, and 40% booting fertilizer. In 2022, the biogas slurry was applied as a tillering fertilizer, and in 2023, the biogas slurry was applied as a base fertilizer. The application of N, P, and K fertilizers was consistent with the optimal fertilization treatment. All fertilizers were distributed manually, and the management measures of the field plot were consistent with local practices.

The rice variety planted was Wankenuo No. 2, and plant space was 30 cm × 14 cm, with two or three seedlings in each hole. The specific application times of base fertilizer, tillering fertilizer, booting fertilizer, and rice harvest time are shown in Table 2.

### 2.3. Collection and Analysis of Soil and Plant Samples

Before the experiment and after rice harvest, fresh soil samples were collected from the 0–20 cm layer by soil drilling in each plot according to the five-point sampling method. After fully mixing, a portion of the soil samples was stored at 4 °C. The other portion of the soil samples were air-dried and screened with 2 and 0.15 mm sieves. The methods for determining the physical and chemical properties of the soil are shown in Table 3.

During the mature stage of rice, straw and grain yields were measured separately in each plot. Plant samples from each plot were collected at multiple points using the double diagonal method to determine plant height, 1000-grain weight, and kernels per spike. The samples were threshed separately according to straw and grain and then crushed for N, P, and K content analysis in grain and straw. The N, P, and K contents in plants were determined using the Kellner method, molybdenum yellow colorimetry, and flame spectrophotometry, respectively.

### 2.4. Calculations and Statistical Analysis

N (P and K) uptake in rice grain was calculated by the product of rice grain yield and N (P and K) content. N (P and K) uptake of rice straw was calculated by the product of rice straw yield and straw N (P and K) uptake content. The fertilizer utilization efficiency was determined by the difference in N (P and K) uptake between rice grain and straw in fertilized and non-fertilized areas, and the ratio of the N (P and K) application amount.

(1) $${N\;(P\;\mathrm{and}\;K)\mathrm{uptake}\;\mathrm{in}\;\mathrm{rice}\;\mathrm{grain}\;(\mathrm{NUG},\;\mathrm{PUG},\;\mathrm{KUG},\;\mathrm{kg}\text{·}ha}^{-1})=GY\times GN(GP,GK)$$

(2) $${N\;(P\;\mathrm{and}\;K)\mathrm{uptake}\;\mathrm{of}\;\mathrm{rice}\;\mathrm{straw}\;(\mathrm{NUS},\;\mathrm{PUS},\;\mathrm{KUS},\;\mathrm{kg}\text{·}\mathrm{ha}}^{-1})=SY\times SN\;(SP,SK)$$

(3) $$N\;\mathrm{utilization}\;\mathrm{efficiency}\;(\mathrm{NUE},\;\%)=\frac{\left(NUG+NUS)-({NUG}_{0}+{NUS}_{0}\right)}{TNA}\times 100$$

(4) $$P\;\mathrm{utilization}\;e\mathrm{fficiency}\;(\mathrm{PUE},\;\%)=\frac{\left(PUG+PUS)-({PUG}_{0}+{PUS}_{0}\right)}{TPA}\times 100$$

(5) $$K\;\mathrm{utilization}\;e\mathrm{fficiency}\;(\mathrm{KUE},\;\%)=\frac{\left(KUG+KUS)-({KUG}_{0}+{KUS}_{0}\right)}{TKA}\times 100$$

*GY* and *SY* refer to the rice grain yield and straw yield (kg·ha^−1^); *GN* and *SN* refer to N content of rice grain and straw (%); *GP* and *SP* refer to P content of rice grain and straw (%); *GK* and *SK* refer to K content of rice grain and straw (%); *NUG*, *NUS* (*PUG*, *PUS*, *KUG*, *KUS*) refer to N (P, K) uptake by rice grains and straws in fertilized area (kg·ha^−1^); *NUG*_0_, *NUS*_0_ (*PUG*_0_, *PUS*_0_, *KUG*_0_, *KUS*_0_) refer to N (P, K) uptake by rice grains and straws in non-fertilized area (kg·ha^−1^); *TNA* (*TPA*, *TKA*) refer to total N (P, K) application (kg·ha^−1^).

The IBM-SPSS Statistics 23 software package (SPSS, Chicago, IL, USA) was used to perform a one-way analysis of variance (LSD) between processing treatments, and *p* < 0.05 was considered a significant difference. Origin 2023 (Systat Software, Inc., Washington, DC, USA) software was used for mapping.

## 3. Results

### 3.1. Analysis of the Nutrient Content of Biogas Slurries in Lujiang County

**Figure 1:**
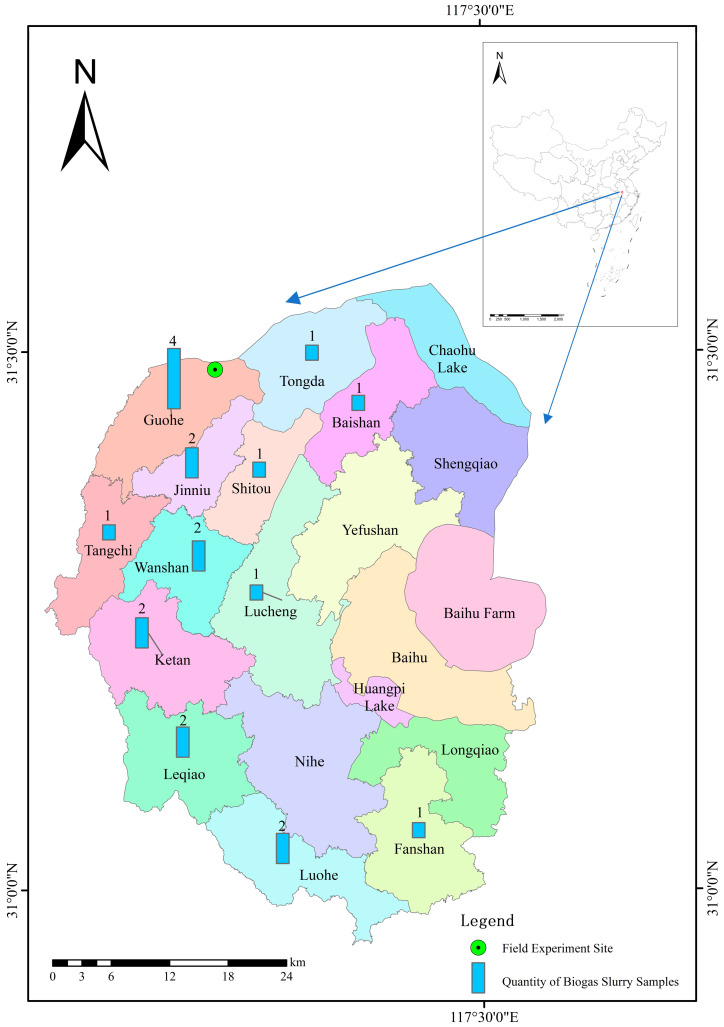
Field experiment site, location, and quantity of biogas slurry samples.

The nutrient content of biogas slurries sampled from 12 townships of Lujiang County (including Guohe Town, Jinniu Town, Ketan Town, and so on) in 2022 and 2023 was analyzed. The field experiment site, location, and quantity of biogas slurry sampling are shown in Figure 1.

**Figure 2:**
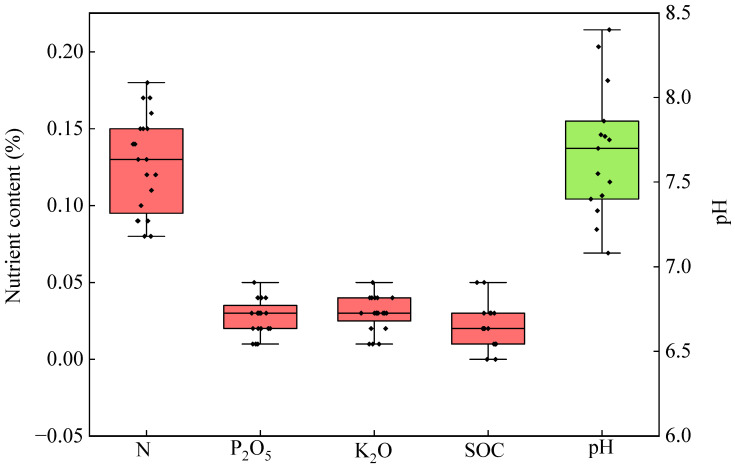
Analysis of nutrient content of biogas slurry in twelve townships of Lujiang County.

As shown in Figure 2, the N content of the biogas slurry was 0.08–0.18%, and the P and K contents were 0.01–0.05%. The soil organic C (SOC) was 0.01–0.05%, and the pH was 7.08–8.40.

### 3.2. Rice Yield

**Figure 3:**
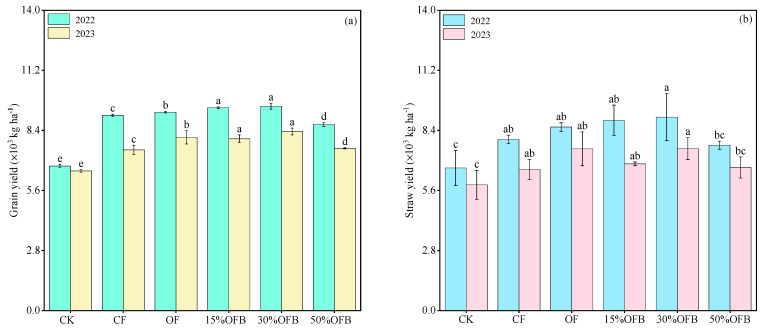
Effects of biogas slurry substitution for fertilizer on grain yield (**a**) and straw yield (**b**) of rice. Bars represent the SD of the means (*n* = 3), and the different lowercase letters above the column indicate that the differences among treatments are significant at *p* < 0.05.

The grain and straw yields of rice in 2022 were 6737.39–9527.44 and 6654.98–9019.71 kg·ha^−1^, respectively (Figure 3). In 2023, rice grain and straw yields were 6508.60–8346.14 and 5857.97–7548.34 kg·ha^−1^, respectively, and the rice grain yield under all treatments was higher in 2022 than in 2023. Compared with CF treatment, grain and straw yields of OF treatment increased in 2022 and 2023, with grain yield increasing by 1.67% and 7.87%, respectively, and straw yield increasing by 7.20% and 14.58%, respectively. In 2022 and 2023, the rice grain yield under 15% OFB and 30% OFB treatments significantly increased (*p* < 0.05) compared with CF treatment. The rice grain yield was the highest after 30% OFB treatment in two years. In 2022, the grain yield of 50% OFB treatment was significantly lower than that of CF treatment (*p* < 0.05) by 4.52%, while there was no significant difference between 50% OFB treatment and CF treatment in 2023. In comparison to the OF treatment, the yields of rice grain and straw showed an increasing trend in both the 15% OFB and 30% OFB treatments during 2022. Furthermore, in 2023, only the 30% OFB treatment led to an elevation in both rice grain and straw yields. With the increase in the proportion of biogas slurry replacing fertilizer, the rice yield in 2022 and 2023 showed a trend of first increasing and then decreasing (30% OFB > 15% OFB > 50% OFB).

In 2022 and 2023, the plant height, 1000-grain weight, and kernels per spike of rice under the OF treatment increased compared with CF treatment, but the differences were not statistically significant (Table 4). Compared with CF treatment, the plant height, 1000-grain weight, and kernels per spike of rice treated with 15% OFB, 30% OFB, and 50% OFB increased in 2022 and 2023. The 1000-grain weight and kernels per spike of rice treated with 30% OFB showed significant differences (*p* < 0.05), with the 1000-grain weight increasing by 25.72% and 6.82% and kernels per spike increasing by 61.19% and 36.81%, respectively. Compared with OF treatment, the 1000-grain weight of rice treated with 30% OFB in 2022 and 2023 significantly increased (*p* < 0.05) by 25.72% and 6.50%, respectively. With the increase in the proportion of biogas slurry replacing fertilizer, the composition factors of rice yield in 2022 and 2023 showed a trend of first increasing and then decreasing.

### 3.3. Fertilizer Utilization Efficiency

Compared with CF treatment, the N, P, and K uptake of rice grains under OF treatment increased by 12.83%, 4.77%, and 33.20%, respectively, in 2022 and by 4.46%, 23.96%, and 7.64%, respectively, in 2023 (Table 5). In contrast to CF treatment, the N uptake of rice straws under OF treatment increased by 5.50% and 3.90%, the P uptake increased by 15.76% and 27.89%, and the K uptake increased by 8.78% and 45.65% in 2022 and 2023, respectively. Compared with CF treatment, in 2022, 15% OFB and 30% OFB treatments significantly increased (*p* < 0.05) the N, P, and K uptake of rice grains, and the 30% OFB treatment significantly increased (*p* < 0.05) the N, P, and K uptake in rice straw. In 2023, the N, P, and K uptake of rice grains and straws treated with 15% OFB, 30% OFB, and 50% OFB were significantly increased (*p* < 0.05) compared with those treated with CF. When compared to OF treatment, in 2022 and 2023, 30% OFB treatment significantly increased (*p* < 0.05) the N, P, and K uptake in grains and straws, and the 15% OFB and 50% OFB treatments significantly increased (*p* < 0.05) the K uptake of grain and straw. With the increase in the proportion of biogas slurry replacing fertilizer, the N, P, and K uptake in grain and straw in 2022 and 2023 showed a trend of first increasing and then decreasing (30% OFB > 15% OFB > 50% OFB).

**Figure 4:**
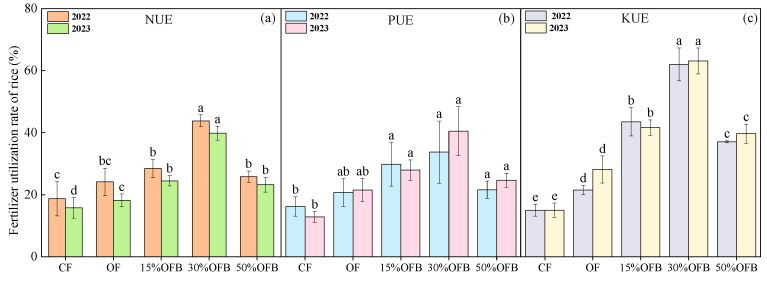
Effects of biogas slurry on N use efficiency (NUE) (**a**), P use efficiency (PUE) (**b**) and K use efficiency (KUE) (**c**) of rice. Bars represent the SD of the means (*n* = 3). The different lowercase letters above the column indicate that the differences among treatments are significant at *p* < 0.05.

Compared with CF treatment, the N use efficiency (NUE), P use efficiency (PUE), and K use efficiency (KUE) of rice under OF treatment increased by 29.11%, 27.44%, and 43.31% in 2022, and by 15.10%, 67.71%, and 87.34% in 2023, respectively (Figure 4). The NUE, PUE, and KUE of rice treated with 15% OFB, 30% OFB, and 50% OFB were significantly increased (*p* < 0.05) compared with CF treatment. In contrast to OF treatment, the NUE and KUE of rice treated with 15% OFB, 30% OFB, and 50% OFB were significantly increased (*p* < 0.05). With the increase in the proportion of biogas slurry replacing fertilizer, the NUE, PUE, and KUE in rice in 2022 and 2023 showed a trend of first increasing and then decreasing (30% OFB > 15% OFB > 50% OFB).

### 3.4. Soil Nutrients

Compared with CF treatment, the contents of soil total N (TN), NH_4_^+^-N, NO_3_^−^-N, and hydrolysable N (HN) under OF treatment in 2022 increased by 1.87%, 6.25%, 26.85%, and 18.50%, respectively (Table 6). In 2023, the content of NH_4_^+^-N, NO_3_^−^-N, and HN in soil under OF treatment increased by 16.33%, 14.13%, and 2.29%, respectively. In 2022, the contents of NH_4_^+^-N and HN in soil under 30% OFB treatment significantly increased (*p* < 0.05) compared with CF treatment, showing increases of 16.03% and 32.86%, respectively. In contrast to CF treatment, the contents of NH_4_^+^-N, NO_3_^−^-N, and HN increased under 15% OFB, 30% OFB, and 50% OFB treatments in 2023, while the contents of TN, NH_4_^+^-N, and HN under 30% OFB treatment significantly increased (*p* < 0.05) by 31.00%, 38.90%, and 21.80%, respectively. There were no significant differences in soil NO_3_^−^-N content between CF and other treatments. Over the course of two years, the soil TN content increased under 15% OFB, 30% OFB, and 50% OFB treatments when compared to the OF treatment. Additionally, the soil NH_4_^+^-N content rose under 15% OFB and 30% OFB treatments as well. Compared with OF treatment, in 2022, the soil NH_4_^+^-N and HN contents significantly increased (*p* < 0.05) by 9.21% and 12.12%, respectively, under 30% OFB treatment. When compared to OF treatment, in 2023, the contents of TN and HN significantly increased by 35.05% and 19.07%, respectively, under 30% OFB treatment (*p* < 0.05). With the increase in the proportion of biogas slurry replacing chemical fertilizer, the contents of NH_4_^+^-N and HN in the soil first increased and then decreased (30% OFB > 15% OFB > 50% OFB) in 2022 and 2023.

Compared with CF treatment, OF treatment increased the soil bulk density by 7.08% and 6.48%, pH by 0.76% and 4.89%, SOC by 1.28% and 0.20%, and available P by 19.16% and 2.53% in 2022 and 2023, respectively (Table 7). The available K was reduced by 21.56% and 3.07% in 2022 and 2023, respectively. In 2022, the contents of SOC, available K, and P under 30% OFB treatment significantly increased (*p* < 0.05) by 14.77%, 43.10%, and 17.67%, respectively, compared with CF treatment. In 2023, soil available K and SOC contents under 15% OFB and 30% OFB treatments significantly increased (*p* < 0.05). In contrast to OF treatment, the SOC and available K contents increased under 15% OFB, 30% OFB, and 50% OFB treatments in two years, and the available P content also increased under 15% OFB and 30% OFB treatments. The 15% OFB, 30% OFB, and 50% OFB treatments significantly increased (*p* < 0.05) the soil available K content in 2022 and 2023, and the 15% OFB and 30% OFB treatments significantly increased (*p* < 0.05) the SOC content in 2023. With the increase in the proportion of biogas slurry replacing fertilizer, the contents of SOC, available P, and K showed a trend of first increasing and then decreasing (30% OFB > 15% OFB > 50% OFB) in 2022 and 2023.

### 3.5. Correlation Analysis of Yield, Soil Fertility and Fertilizer Utilization Efficiency

**Figure 5:**
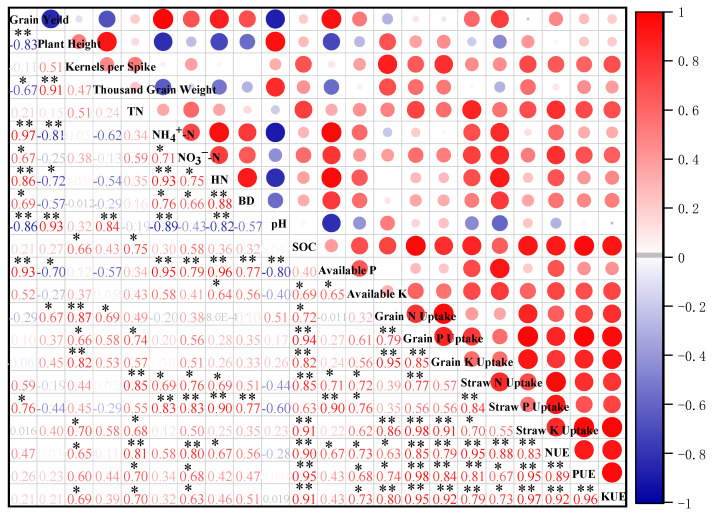
Correlation analysis of yield and yield composition, soil nutrient content and nutrient uptake of rice grain and straw, fertilizer utilization efficiency. Note: * indicates significant correlation (*p* < 0.05), ** indicates significant correlation (*p* < 0.01).

As shown in Figure 5, grain yield was significantly positively correlated with soil NH_4_^+^-N, hydrolysable N, and available P contents (*p* < 0.01), significantly positively correlated with soil NO_3_^−^-N, bulk density (BD), and straw P uptake (*p* < 0.05), significantly negatively correlated with rice plant height and soil pH (*p* < 0.01), and significantly negatively correlated with kernels per spike (*p* < 0.05). There was a significant positive correlation between SOC and N, P, and K utilization efficiency (*p* < 0.01). Soil TN and NO_3_^−^-N contents were significantly positively correlated with the NUE, PUE, and KUE (*p* < 0.05).

## 4. Discussion

### 4.1. Effects of Biogas Slurry Substitution for Fertilizer on Rice Yield

In this study, the rice grain yield was 6737.39–9527.44 and 6508.60–8346.14 kg·ha^−1^ in 2022 and 2023, respectively. The overall rice yield in 2023 was lower than that in 2022. The reason may be that the extreme maximum temperature from June to August in Anhui province was 36–38 °C in 2023. Affected by Typhoon “Du Suri” in late July, there was too much rainfall, the rice seed setting rate decreased, and rice was vulnerable to diseases and pests. Moreover, there was less sunshine during the whole growing period of rice, resulting in a lower rice yield. This resulted in poor environmental conditions, including temperature, rainfall, and sunshine time, in Hefei compared with 2022 [18,19], and they were not suitable for rice growth [20]. In this experiment, compared with CF treatment, OF treatment significantly increased rice yield in 2022 and 2023 (*p* < 0.05), indicating that optimized N and K application can effectively increase rice yield. Studies have applied 10%, 20%, and 40% N fertilizer at the booting stage, resulting in rice yields of 5494.50, 10,400, and 5002.73 kg·ha^−1^, respectively, which indicated that 20% N fertilizer applied at the booting stage was conducive to increasing rice yield [21,22,23]. In this study, the proportion of N fertilizer applied at the booting stage increased from 15% in the CF treatment to 20% in the OF treatment, which was one of the reasons why the optimized fertilization treatment resulted in a higher rice yield than the conventional fertilization treatment. The results of previous experiments showed that when the proportion of K fertilizer applied at the booting stage was 40% and 30%, the rice yield was 5002.73 and 3724 kg·ha^−1^, respectively [23,24]. In addition, K fertilizer applied at the tillering stage can promote N and P uptake, increase lodging resistance and the seed setting rate of rice, and further increase the yield, thus increasing the K fertilizer utilization efficiency [25]. In the present study, K fertilizer was applied as 45% base fertilizer, 15% tillering fertilizer, and 40% booting fertilizer under OF treatment, which was further optimized on the basis of conventional fertilization (50% base fertilizer + 50% booting fertilizer). Therefore, adjusting the proportion of K fertilizer at the booting stage and increasing the proportion of K fertilizer at the tillering stage may be another reason for optimizing fertilization to increase yield and fertilizer utilization efficiency.

Organic fertilizer instead of chemical fertilizer can improve the ability of rice to absorb and transform nutrients, increasing nutrient accumulation and thus achieving high yield [26,27]. When organic fertilizers replaced 15%, 30%, and 50% of fertilizer, the rice yield showed a trend of first increasing and then decreasing, and the yield effect was the best in the 30% replacement treatment [28], which was consistent with the results of this experiment. In this study, the yield of rice treated with 15% and 30% of fertilizer replaced by biogas slurry was significantly higher than that of rice treated with conventional fertilizer, and the yield of rice treated with 30% fertilizer replacement with biogas slurry was the highest. Biogas slurries were rich in N, P, K, and other nutrients required for crop growth, which can meet the needs of soil microorganisms, improve the richness of the soil microbial community, reduce soil water and nutrient losses, promote crop growth, and enhance the root absorption capacity [29]. In this study, the N, P, and K contents of grains and stalks treated with 15% and 30% of fertilizer replacement with biogas slurry increased compared with the optimized fertilization treatment. The contents of SOC, NH_4_^+^-N, hydrolysable N, available P and K increased compared with the optimized fertilization treatment, which further confirmed that biogas slurry application promoted rice growth and increased the soil nutrient supply [30]. The biogas slurry partially replaced chemical fertilizer treatment, significantly increased the 1000-grain weight and kernels per spike of rice, and increased crop yield by regulating N release in the early stage and supplementing organic nutrition in the later stage [31]. This study reached a consistent conclusion that the plant height, 1000-grain weight, and kernels per spike of rice treated with 15% and 30% of fertilizer replacement with biogas slurry increased compared with the optimal treatment.

Excessive application of biogas slurry cannot significantly improve crop yield because it releases nutrients slowly. Soil microorganisms need time to decompose it, and the nutrients available in soil cannot timely meet the early growth needs of crops, resulting in poor development of vegetative organs, which is not conducive to high and stable crop yield [32]. Compared with the CF treatment, the yield of rice treated with 50% OFB decreased by 4.52% in 2022. Xiao et al. (2023) also showed that wheat yield decreased when 50% of fertilizer was replaced by biogas slurry [14]. This may be because the biogas slurry was applied at the rice tillering stage, and organic fertilizer was not applied at the base fertilizer stage, resulting in insufficient N fertilizer, which affected rice growth. Even if the N content was fully compensated for at the tillering stage, the rice yield was still affected [33]. The nutrient release of biogas slurry organic fertilizer was slow, and an excessive substitution ratio led to an insufficient N supply, affecting rice growth. Therefore, compared with optimal fertilization, rice yield was still reduced [34], and the method by which the biogas slurry was applied may also affect rice yield. Studies have shown that the application of biogas slurry as a base fertilizer can increase yield [30], while application at the tillering stage can reduce yield [35]. In this study, in 2022, when the biogas slurry was applied at the tillering stage, the rice yield under the 50% OFB treatment was significantly lower than that of conventional fertilizer treatment. However, in 2023, when the biogas slurry was applied as a base fertilizer, the rice yield under 50% OFB treatment was not significantly different from that of the conventional fertilizer treatment. This may be because the application of biogas slurry as a base fertilizer can timely provide sufficient nutrients to rice at the tillering stage and promote rice growth and development, while the application of biogas slurry at the tillering stage led to insufficient nutrients for growth in the early growth stage, affecting rice yield [36].

### 4.2. Effects of Biogas Slurry on the Fertilizer Utilization Efficiency of Rice

The N application level can affect the NUE of rice [37], and the NUE decreased with the increase in the N application rate [38]. In this study, the NUE of rice in 2022 and 2023 were 18.69–43.81% and 15.76–39.82%, respectively, which were higher than 17.79–26.61% observed by Liu et al. (2017) [39] and lower than 25.12–48.16% observed by Zhou et al. (2021) [40]. The reason may be that the N application rate in this experiment (225 kg·ha^−1^) was lower than that in Liu et al. (2017) (300 kg·ha^−1^) and higher than that in Zhou et al. (2021) (179.4 kg·ha^−1^) [39,40].

The substitution of organic fertilizer for chemical fertilizer promoted the uptake of N, P, and K in rice grains and straws, and the utilization efficiency of fertilizer increased [41]. In this study, compared with CF treatment, replacing 15%, 30%, and 50% of fertilizer with biogas slurry increased the N, P, and K uptake of rice grains and straws in 2022 and 2023, further significantly improving the fertilizer utilization efficiency (*p* < 0.05). The SOC content was extremely significantly positively correlated with the fertilizer utilization efficiency, and soil TN and NO_3_^−^-N contents were significantly positively correlated with the NUE, PUE, and KUE (Figure 5). This indicated that applying biogas slurry to the soil promoted the mineralization and decomposition of organic matter and other nutrients by soil microorganisms and promoted the release of a large amount of N, P, and K nutrients, which was conducive to the absorption of N, P, and K by crop grains and stalks, thus improving the fertilizer utilization efficiency [42]. In this study, when biogas slurry replaced 15%, 30%, and 50% of fertilizers, the fertilizer utilization efficiency showed a trend of first increasing and then decreasing (30% OFB > 15% OFB > 50% OFB) in 2022 and 2023. The maximum value was found in the 30% OFB treatment, showing a significant effect (*p* < 0.05). Studies have shown that with the increase in the proportion of organic fertilizer replacing chemical fertilizer, the fertilizer utilization efficiency presented a trend of first increasing and then decreasing [43], and the effect of organic fertilizer replacing 30% chemical fertilizer was the best [40]. This was consistent with the results of this study. This may be because the fertilizer effect of organic fertilizer was slow and lasting. When organic fertilizer replaced chemical fertilizer in a proportion that was too high or too low, the available nutrients cannot meet the needs of rice in time. When organic fertilizer replaced 30% of chemical fertilizer, the nutrients required for rice growth and development could be better supplied, and it was easy to absorb N, P, and K [44].

### 4.3. Effects of Biogas Slurry Replacement of Fertilizer on Soil Nutrients

There was a significant positive correlation (*p* < 0.05) between NO_3_^−^-N content in soil and N application amount [45]. In this study, the NO_3_^−^-N content in the 0–20 cm soil layer was 2.51–3.99 mg·kg^−1^, which was higher than 0.48–1.56 mg·kg^−1^ observed by Xiao et al. (2023) in wheat season, possibly because the N application amount was higher in this study (225 kg·ha^−1^) than that in the study of Xiao et al. (2023) (180 kg·ha^−1^) [14]. In this experiment, compared with CF treatment, replacing 15%, 30%, and 50% of fertilizer with biogas slurry increased the contents of SOC, NH_4_^+^-N, and HN. The contents of SOC, NH_4_^+^-N, and hydrolysable N significantly increased when biogas slurry replaced 30% of the fertilizer (*p* < 0.05). Previous studies have also reached a similar conclusion that, compared with conventional treatment, treatment with organic fertilizer instead of chemical fertilizer can increase the contents of soil SOC, NH_4_^+^-N, and hydrolysable N, and the difference was significant when 30% of chemical fertilizer was replaced by organic fertilizer [46,47,48]. The reason may be that organic fertilizer was rich in humic acid and organic acid, which increased the SOC content, provided abundant carbon sources for the soil–crop system [49], and increased the mineralization rate and mineralization potential of N. Furthermore, an increase in SOC content promoted the accumulation of N mineralization amounts [50], increased the content and availability of soil N, and further increased the content of SOC, NH_4_^+^-N, and hydrolysable N in soil [51].

Compared with the conventional fertilization treatment, the replacement of 15%, 30%, and 50% of fertilizer with biogas slurry increased the available K and P contents in soil, and the replacement of 15% and 30% of fertilizer with biogas slurry significantly increased the available K content in soil (*p* < 0.05). Previous studies have shown that, compared with a single application of chemical fertilizer, biogas slurry, pig manure, commercial organic fertilizer, and cow manure can all increase the soil available K and P contents, and the replacement of 15% and 30% fertilizer with organic fertilizer can significantly increase the soil available K content [46,52,53,54,55]. This was consistent with the results of this study. This may be because a reasonable proportion of organic fertilizer replacing chemical fertilizer promoted the after-effect of P, which was conducive to the conversion of accumulated P in the soil to the available state and significantly increased the P content in the soil [56]. Additionally, the K contained in organic fertilizer was highly mobile, and once the organic fertilizer was applied to the soil, it decomposed rapidly, released more organic P, and increased the P and K contents in the soil [57].

## 5. Conclusions

This study investigated whether optimizing N and K topdressing methods increased rice yield and fertilizer utilization efficiency and improved soil fertility. The substitution of biogas slurry for N fertilizer increased rice yield and yield composition, and replacing 30% N fertilizer with biogas slurry had the highest yield of grain and straw in rice. In addition, the substitution of biogas slurry for N fertilizer promoted the uptake of N, P, and K in rice grains and straws and improved the fertilizer utilization efficiency. Replacing fertilizer with biogas slurry increased the contents of SOC, NH_4_^+^-N, hydrolysable N, and available K and improved soil fertility, and the replacement of 30% of N fertilizer with biogas slurry had a more obvious effect. The 30% OFB treatment resulted in the highest rice yield and fertilizer utilization efficiency and improved soil fertility, indicating that biogas slurry replacing 30% of fertilizer was the best application mode for rice in this region.

## Figures and Tables

**Table 1:** Fertilizer amount of different treatments in 2022 and 2023 rice seasons.

Years	Treatment	Organic Fertilizer (kg·ha^−1^)	Fertilizer (kg·ha^−1^)			Converted to Pure Nutrients (kg·ha^−1^)		
		Biogas Slurry	Urea	Superphosphate	Potassium Chloride	N	P_2_O_5_	K_2_O
2022	CK	0	0	0	0	0	0	0
	CF	0	485	500	200	225	60	120
	OF	0	485	500	200	225	60	120
	15% OFB	22,500	412	462	189	225	60	120
	30% OFB	45,000	339	425	177	225	60	120
	50% OFB	75,000	242	375	162	225	60	120
2023	CK	0	0	0	0	0	0	0
	CF	0	485	500	200	225	60	120
	OF	0	485	500	200	225	60	120
	15% OFB	30,682	412	372	185	225	60	120
	30% OFB	61,363	339	244	169	225	60	120
	50% OFB	102,272	242	74	149	225	60	120

**Table 2:** Field management time in 2022 and 2023 rice seasons.

Management Measure	2022	2023
Base fertilizer	15 June 2022	9 June 2023
Booting fertilizer	11 July 2022	13 July 2023
Tillering fertilizer	10 August 2022	17 August 2023
Harvest	2 November 2022	15 November 2023

**Table 3:** Soil measurement indexes and methods.

Sample	Measurement Indexes	Measurement Methods
Soil	pH	Potentiometry
	SOC	Potassium dichromate oil bath plus heat capacity method
	TN	Kjeldahl method for nitrogen determination
	NH_4_^+^-N	Indophenol blue colorimetry
	NO_3_^−^-N	Ultraviolet spectrophotometry
	Available P	Sodium bicarbonate extraction-molybdenum antimony resistance spectrophotometry
	Available K	Flame photometer method
	Hydrolysable N	Alkalolytic diffusion method

**Table 4:** Effects of biogas slurry substitution for fertilizer on rice yield components.

Years	Treatment	Plant Height (cm)	Thousand Grain Weight (g)	Kernels per Spike (kernel)
2022	CK	70.53 ± 2.84 b	20.81 ± 0.92 c	124.33 ± 5.13 e
	CF	77.73 ± 2.73 a	22. 51 ± 0.34 b	146.00 ± 3.00 d
	OF	78.40 ± 2.03 a	22. 91 ± 0.44 b	172.67 ± 6.81 c
	15% OFB	79.73 ± 1.30 a	23.05 ± 0.92 b	196.00 ± 5.20 b
	30% OFB	81.80 ± 2.80 a	28.30 ± 0.84 a	235.33 ± 5.69 a
	50% OFB	78.53 ± 1.67 a	22.68 ± 0.42 b	191.00 ± 6.93 b
2023	CK	78.00 ± 4.36 b	23.27 ± 1.32 c	122.00 ± 20.07 b
	CF	90.00 ± 2.00 a	24.53 ± 0.55 bc	144.00 ± 11.53 b
	OF	90.67 ± 1.15 a	24.60 ± 0.66 bc	170.00 ± 34.70 ab
	15% OFB	91.67 ± 2.08 a	25.67 ± 0.32 ab	193.33 ± 8.62 a
	30% OFB	94.00 ± 2.00 a	26.20 ± 0.79 a	197.00 ± 46.60 a
	50% OFB	92.67 ± 1.15 a	24.87 ± 0.15 ab	184.00 ± 40.85 a

Note: Data are presented as mean ± SD (*n* = 3). Different lowercase letters within each column indicate that the differences among treatments are significant at *p* < 0.05.

**Table 5:** Effects of biogas slurry substitution for fertilizer on nutrient uptake of rice.

Years	Treatment	Grain Nutrient Uptake (kg·ha^−1^)			Straw Nutrient Uptake (kg·ha^−1^)		
		N	P	K	N	P	K
2022	CK	51.28 ± 9.34 d	10.05 ± 2.91 d	6.26 ± 1.09 e	28.12 ± 8.76 c	7.43 ± 1.05 d	22.33 ± 2.85 d
	CF	77.54 ± 7.98 c	16.77 ± 1.00 c	15.18 ± 1.46 d	41.67 ± 4.83 b	9.39 ± 2.00 cd	31.42 ± 1.32 c
	OF	87.49 ± 9.66 bc	17.57 ± 0.51 bc	20.22 ± 3.04 c	43.96 ± 1.32 b	10.87 ± 0.23 bc	34.18 ± 1.25 c
	15% OFB	94.05 ± 8.34 b	20.45 ± 1.44 ab	26.86 ± 0.57 b	47.07 ± 4.34 b	12.80 ± 1.34 b	53.94 ± 5.07 b
	30% OFB	111.89 ± 7.59 a	23.28 ± 0.46 a	35.22 ± 2.12 a	63.84 ± 3.38 a	17.27 ± 0.81 a	67.76 ± 5.92 a
	50% OFB	90.25 ± 2.30 bc	18.90 ± 1.95 bc	23.69 ± 1.56 b	44.99 ± 2.39 b	11.65 ± 0.75 b	49.38 ± 1.68 b
2023	CK	75.29 ± 1.60 e	11.89 ± 1.27 d	14.50 ± 1.43 c	18.77 ± 3.91 d	5.45 ± 0.75 e	23.36 ± 5.50 e
	CF	95.68 ± 3.14 d	15.36 ± 0.89 cd	22.77 ± 1.38 b	27.98 ± 4.27 c	6.97 ± 0.46 d	33.10 ± 1.42 d
	OF	99.95 ± 5.62 cd	19.04 ± 2.15 bc	23.40 ± 1.34 b	29.07 ± 1.35 c	8.50 ± 0.91 c	48.21 ± 3.98 c
	15% OFB	106.58 ± 2.34 b	21.58 ± 1.88 b	30.28 ± 2.51 a	36.68 ± 1.94 b	9.81 ± 0.14 b	57.53 ± 2.72 b
	30% OFB	111.82 ± 0.74 a	27.16 ± 3.92 a	33.23 ± 4.77 a	65.97 ± 4.79 a	11.76 ± 1.01 a	80.34 ± 7.10 a
	50% OFB	104.40 ± 0.87 bc	20.63 ± 0.96 b	29.03 ± 0.22 a	36.05 ± 5.21 b	8.82 ± 0.49 bc	56.43 ± 3.55 b

Note: Data are presented as mean ± SD (*n* = 3). Different lowercase letters within each column indicate that the differences among treatments are significant at *p* < 0.05.

**Table 6:** Effects of biogas slurry replacing fertilizer on soil N content.

Years	Treatment	TN (g·kg^−1^)	NH_4_^+^-N (mg·kg^−1^)	NO_3_^−^-N (mg·kg^−1^)	HN (mg·kg^−1^)
2022	CK	0.97 ± 0.11 b	33.19 ± 2.63 c	2.51 ± 0.54 b	90.90 ± 3.10 c
	CF	1.07 ± 0.08 ab	42.10 ± 0.66 b	2.98 ± 0.62 ab	92.83 ± 7.95 c
	OF	1.09 ± 0.07 ab	44.73 ± 3.45 b	3.78 ± 0.49 a	110.00 ± 5.29 b
	15% OFB	1.02 ± 0.02 b	45.15 ± 1.05 b	3.55 ± 0.31 ab	112.67 ± 3.51 ab
	30% OFB	1.23 ± 0.10 a	48.85 ± 0.85 a	3.99 ± 0.57 a	123.33 ± 3.21 a
	50% OFB	1.06 ± 0.12 b	42.67 ± 2.02 b	3.15 ± 0.72 ab	111.13 ± 11.04 b
2023	CK	0.95 ± 0.03 c	23.97 ± 2.97 c	2.60 ± 0.44 b	76.77 ± 4.26 b
	CF	1.00 ± 0.04 c	27.25 ± 6.71 bc	2.83 ± 0.43 ab	77.30 ± 8.69 b
	OF	0.97 ± 0.03 c	31.70 ± 2.20 abc	3.23 ± 0.34 ab	79.07 ± 3.71 b
	15% OFB	0.99 ± 0.01 c	32.95 ± 4.25 ab	3.12 ± 0.52 ab	91.87 ± 4.14 a
	30% OFB	1.31 ± 0.07 a	37.85 ± 2.95 a	3.60 ± 0.44 a	94.15 ± 1.35 a

Note: Data are presented as mean ± SD (*n* = 3). Different lowercase letters within each column indicate that the differences among treatments are significant at *p* < 0.05. TN: total N; HN: hydrolysable N.

**Table 7:** Effects of biogas slurry replacing fertilizer on soil nutrients.

Years	Treatment	BD (g·cm^−3^)	pH (H_2_O)	SOC (g·kg^−1^)	Available P (mg·kg^−1^)	Available K (mg·kg^−1^)
2022	CK	1.22 ± 0.04 a	5.50 ± 0.03 a	9.40 ± 1.08 c	29.93 ± 3.72 b	79.67 ± 9.50 c
	CF	1.13 ± 0.00 b	5.29 ± 0.05 c	10.17 ± 0.79 bc	30.63 ± 4.90 b	111.33 ± 3.21 b
	OF	1.21 ± 0.03 a	5.33 ± 0.05 c	10.30 ± 0.90 abc	36.50 ± 2.52 ab	87.33 ± 9.71 c
	15% OFB	1.23 ± 0.02 a	5.44 ± 0.05 ab	11.02 ± 0.25 ab	39.07 ± 9.23 ab	127.67 ± 7.77 a
	30% OFB	1.23 ± 0.06 a	5.27 ± 0.07 c	11.75 ± 0.90 a	43.83 ± 2.41 a	131.00 ± 9.85 a
	50% OFB	1.24 ± 0.03 a	5.36 ± 0.07 bc	10.48 ± 0.23 abc	32.33 ± 2.73 b	111.67 ± 2.52 b
2023	CK	1.16 ± 0.08 a	6.57 ± 0.32 a	9.71 ± 0.44 c	21.67 ± 5.33 a	65.00 ± 5.00 d
	CF	1.08 ± 0.10 a	6.34 ± 0.35 a	10.11 ± 0.38 c	22.50 ± 7.37 a	87.00 ± 7.00 c
	OF	1.15 ± 0.03 a	6.65 ± 0.39 a	10.13 ± 0.52 c	23.07 ± 2.64 a	84.33 ± 4.04 c
	15% OFB	1.19 ± 0.07 a	6.53 ± 0.45 a	11.19 ± 0.24 b	27.83 ± 1.59 a	116.67 ± 5.77 a
	30% OFB	1.16 ± 0.12 a	6.31 ± 0.38 a	12.59 ± 0.91 a	29.77 ± 2.83 a	117.67 ± 7.09 a
	50% OFB	1.15 ± 0.03 a	6.27 ± 0.39 a	10.48 ± 0.38 bc	22.63 ± 5.59 a	102.00 ± 2.00 b

Note: Data are presented as mean ± SD (*n* = 3). Different lowercase letters within each column indicate that the differences among treatments are significant at *p* < 0.05. BD: bulk density, SOC: soil organic C.

## Data Availability

The data presented in this study are available on request from the corresponding author.
